# Second-Generation Antimitotics in Cancer Clinical Trials

**DOI:** 10.3390/pharmaceutics13071011

**Published:** 2021-07-02

**Authors:** Pedro Novais, Patrícia M. A. Silva, Isabel Amorim, Hassan Bousbaa

**Affiliations:** 1 CESPU, Institute of Research and Advanced Training in Health Sciences and Technologies (IINFACTS), Rua Central de Gandra, 1317, 4585-116 Gandra, Portugal; pedro.ha.novais@gmail.com (P.N.); patricia.silva@cespu.pt (P.M.A.S.); 2 Faculty of Sciences, University of Porto, Rua do Campo Alegre, s/n, 4169-007 Porto, Portugal; 3 ICBAS, Instituto de Ciências Biomédicas Abel Salazar, University of Porto, 4050-313 Porto, Portugal; 4 GreenUPorto (Sustainable Agrifood Production) Research Center, Faculty of Sciences, University of Porto, Rua do Campo Alegre, s/n, 4169-007 Porto, Portugal; mpamorim@fc.up.pt

**Keywords:** cancer, antimitotics, mitotic slippage, spindle assembly checkpoint, clinical trials

## Abstract

Mitosis represents a promising target to block cancer cell proliferation. Classical antimitotics, mainly microtubule-targeting agents (MTAs), such as taxanes and vinca alkaloids, are amongst the most successful anticancer drugs. By disrupting microtubules, they activate the spindle assembly checkpoint (SAC), which induces a prolonged delay in mitosis, expected to induce cell death. However, resistance, toxicity, and slippage limit the MTA’s effectiveness. With the desire to overcome some of the MTA’s limitations, mitotic and SAC components have attracted great interest as promising microtubule-independent targets, leading to the so-called second-generation antimitotics (SGAs). The identification of inhibitors against most of these targets, and the promising outcomes achieved in preclinical assays, has sparked the interest of academia and industry. Many of these inhibitors have entered clinical trials; however, they exhibited limited efficacy as monotherapy, and failed to go beyond phase II trials. Combination therapies are emerging as promising strategies to give a second chance to these SGAs. Here, an updated view of the SGAs that reached clinical trials is here provided, together with future research directions, focusing on inhibitors that target the SAC components.

## 1. Introduction

**Figure 1:**
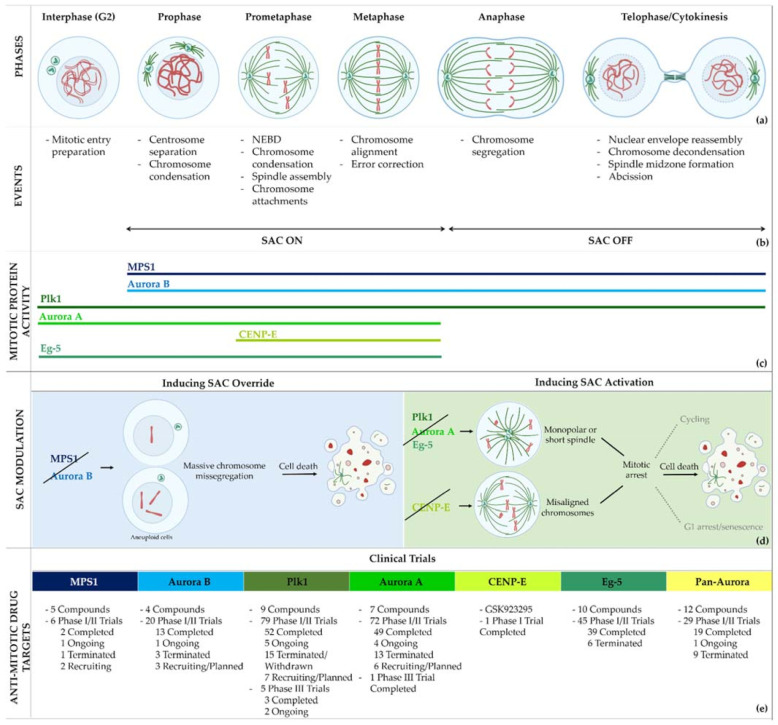
Targeting mitosis for cancer treatment. (**a**,**b**) Representation of G2 interphase and the stages of mitosis. A description of the main cellular changes at each stage is presented. The progression throughout mitosis is monitored by the spindle assembly checkpoint activity (SAC ON and SAC OFF). (**c**) Activity of mitotic proteins during G2 and mitotic phases. MPS1, Aurora B, and PLK1 kinases are involved in several processes, being activated from G2 of interphase to telophase/cytokinesis. Aurora A and Eg-5 proteins ensure proper bipolar spindle shape, remaining activated from G2 to metaphase. CENP-E is required for accurate kinetochore–microtubule attachments, operating from prometaphase to metaphase. (**d**) SAC modulation by targeting mitotic proteins. MPS1 and Aurora B inhibition leads to SAC override, followed by massive chromosome missegregation and cell death. PLK1, Aurora A and Eg-5 inhibition induces spindle defects, while CENP-E inhibition promotes chromosome misalignment, leading to SAC activation, which in turn arrests cells in mitosis. Under mitotic arrest, the cell undergoes death or alternative pathways, namely G1 arrest/senescence, or continues cycling. (**e**) Second-generation antimitotics in clinical trials. Summary of antimitotic drug targets in different phases of clinical trials and current status. Created in BioRender.

The cell cycle is a tightly regulated process in which a parental cell gives rise to two genetically identical daughter cells. Cell cycle progression is under the control of the family of serine/threonine kinases cyclin-dependent kinases (Cdk 1, 2, 4, and 6) and their regulatory subunits cyclins (A, B, D, and E). While Cdks’ concentration is constant throughout the cell cycle, their activation depends on the oscillation of cyclin levels at different phases of the cell cycle [1]. The cell cycle is divided into two phases, interphase and mitosis. Interphase is a time of synthesis and growth, occurring according to the consecutive phases G1, S, and G2, during which the DNA is replicated. Mitosis consists of five active phases: prophase, prometaphase, metaphase, anaphase, and telophase, followed by cytokinesis. During prophase, chromosomes start to condense and the centrosomes start to migrate to the opposite sides of the mitotic cell. After nuclear envelope breakdown (NEBD), at the onset of prometaphase, microtubules emanating from centrosomes grow to assemble the mitotic spindle and capture the chromosomes by attaching to their kinetochores. Chromosomes then align at the spindle equator, forming the metaphase plate. When all chromosomes are bipolarly attached to spindle microtubules, sister chromatids are separated and segregate at the anaphase. The nuclear envelope reassembles at the telophase and the cytoplasm divides (cytokinesis), giving rise to two genetically equal daughter cells (Figure 1a,b).

**Figure 2:**
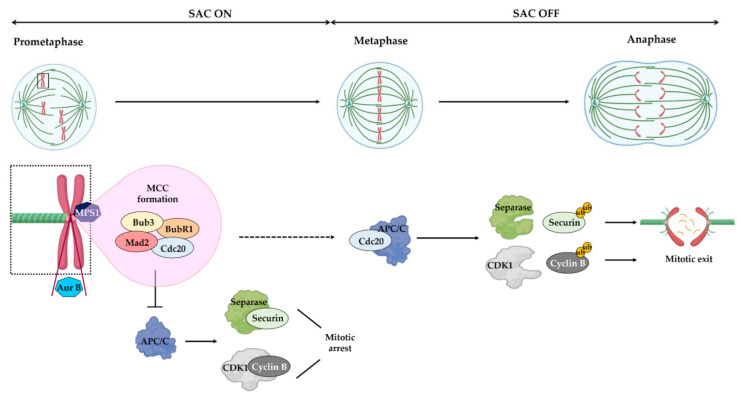
Spindle assembly checkpoint mechanism. In response to unattached or improperly attached kinetochores (Prometaphase), the SAC is turned ON and promotes the assembly of the mitotic checkpoint complex (MCC), made of Mad2, Bub3, BubR1 and Cdc20. At these kinetochores, MPS1 recruits Bub3, Bub1 and BubR1. The MCC inhibits the activity of anaphase-promoting complex/cyclosome (APC/C), leading to the stabilization of separase/securin and CDK1/cyclin B complexes, and consequent mitotic arrest. The Aurora B kinase (Aur B), associated with centromere heterochromatin, promotes proper kinetochore–microtubule attachments. Once all chromosomes are properly attached to spindle microtubules and are aligned at the metaphase plate (metaphase), the SAC is turned OFF, through MCC disassembly, and consequently Cdc20 can bind and activates the APC/C, resulting in the ubiquitylation (ub) of cyclin B and securin mitotic substracts. In turn, separase can cleave cohesins to promote sister chromatid separation (anaphase), while Cdk1 inactivation promotes exit from mitosis. Created in BioRender.

A successful mitosis relies on equal chromosome segregation at the transition from metaphase to anaphase. Kinetochore–microtubule attachment defects lead to the missegregation of chromosomes resulting in genome instability, a hallmark of cancer [2,3]. Fortunately, the fidelity of chromosome segregation is ensured by the spindle assembly checkpoint (SAC) (Figure 2). The SAC consists of a protein network that delays the anaphase in the presence of erroneous kinetochore–microtubule attachments or the absence of attachments [4]. SAC activation is mediated by the orderly orchestrated recruitment to the unattached kinetochores of the SAC proteins monopolar spindle 1 (Mps1), Aurora kinase B, budding uninhibited by benomyl 1 (Bub1), and mitotic arrest deficiency 1 (Mad1). Consequently, the mitotic checkpoint complex (MCC) is assembled, which is formed by Mad2, Bub1-related 1 (BubR1), Bub3 and Cdc20. By sequestering Cdc20, the MCC inhibits the ultimate target of SAC, the anaphase promoting complex/cyclosome (APC/C), an E3 ubiquitin ligase. When all kinetochores are correctly attached to microtubules, the SAC is turned off and APC/C becomes active, targeting securin and cyclin B for proteolysis. The degradation of securin releases the protease separase, which thus cleaves the cohesin rings, allowing sister chromatids to separate, whereas the proteolysis of cyclin B triggers mitotic exit [4].

Based on the uncontrolled proliferation of many cancers, anticancer drugs have been developed to block the cell cycle, particularly mitosis. In this review, we will briefly discuss the current antimitotic approaches, and focus on the new generation of promising antimitotics that have reached clinical trials, with particular emphasis on their clinical efficacy. These so-called second-generation antimitotics (SGAs) target the mitotic kinases and spindle motor proteins. Possible research directions will be discussed.

## 2. Limitations of Current Microtubule-Targeting Agents

Microtubule-targeting agents (MTAs) are the main current antimitotic drugs in the clinic, and are widely used for the treatment of several cancers [5]. MTAs are divided into two groups, based on their action mechanism: microtubule destabilizers, such as the vinca alkaloids, that inhibit microtubule polymerization; and microtubule stabilizers, such as taxanes, that enhance microtubule polymerization [6]. Both classes impair a functional mitotic spindle, leading to SAC activation and subsequent mitotic arrest, which usually results in cell death by apoptosis [7]. However, other outcomes are possible after MTA treatment. After a prolonged mitotic arrest, cells may exit mitosis without undergoing cytokinesis, originating tetraploid cells, a process known as mitotic slippage, which results from a constant and slow degradation of cyclin B even when SAC is on [8]. The slipped cells can follow three possible fates: become senescent, undergo post-mitotic death, or continue dividing [9]. Therefore, mitotic slippage, together with efflux pumps, mutations in tubulin genes, and deficient apoptotic signaling, represent the main reasons for the therapeutic failure of MTAs [10]. Additionally, MTAs treatment is also frequently associated with neurological and myeloid toxicity [10].

## 3. Second-Generation Antimitotics in Clinical Trials

Due to the aforementioned limitation of MTAs, alternative approaches to directly targeting microtubules were developed to block cells in mitosis. These new strategies consist of inhibiting the activity of mitotic proteins, especially kinases and motor proteins, that play crucial roles in different processes during mitosis, such as mitotic entry, spindle assembly, chromosome congression, or SAC regulation. The inhibition of these proteins is made possible through small molecules or small interfering RNAs (siRNAs), known as the second-generation antimitotics (SGAs), with promising outcomes in preclinical assays [11]. Here, we will focus on those SGAs that reached clinical trials, namely, inhibitors of Mps1, polo-like kinase 1 (Plk1), Aurora kinases, Eg-5, and centromeric protein E (CENP-E), review their clinical outcomes, and provide future research directions.

### 3.1. Mps1

Mps1, also known as TKK, is a dual-specificity protein kinase phosphorylating serine/threonine and tyrosine residues [12]. Mps1 is recruited early in mitosis to unattached kinetochores, where it is responsible for SAC activation through the recruitment of several SAC components to kinetochores and subsequent MCC formation [13]. It has also been involved in DNA damage checkpoint response, chromosome alignment, meiosis, cytokinesis, centrosome duplication, and error-correction of kinetochore–microtubule attachment [14,15,16]. Mps1 is overexpressed in various tumors, correlating with poor prognosis [17]. The inhibition of Mps1 activity compromises the SAC, which leads to premature mitotic exit, resulting in massive aberrant chromosome segregation and subsequent cell death [18,19]. Furthermore, inhibition of Mps1 has been shown to sensitize cancer cells to Paclitaxel [18,20]. Hence, Mps1 became an attractive target for cancer therapy, and a variety of small molecules that inhibit Mps1 have been developed. As with most kinases, Mps1 inhibitors are ATP competitive molecules. So far, five Mps1 inhibitors have been approved to begin clinical trials, namely, BOS 172722, BAY 1217389, BAY1161909, CFI-402257 and S 81694. Six phase I/II studies have been undertaken: two have been completed, one has been terminated, two are recruiting participants and another one is ongoing (Figure 1 and Table 1).

#### 3.1.1. BAY 1217389 and BAY1161909 

BAY 1217389, an Imidazopyridazine, and BAY1161909, a Triazolopyridine, inhibit Mps1 with an IC_50_ of 0.34 nM and 0.63 nM, respectively, in vitro [21]. Both compounds were developed by Bayer and showed similar behavior in vivo, with modest efficacy as single agents. However, both compounds demonstrate synergistic effects when combined with Paclitaxel on the growth inhibition of human tumor xenografts in immunocompromised mice [21]. In the clinical trial involving BAY1161909 (NCT02138812) for the treatment of advanced solid malignancies, in a combinational treatment with 75 mg/m^2^ and 90 mg/m^2^ of Paclitaxel, five (14%) and four (14%) partial responses (PRs) were reported, respectively [22]. However, Bayer decided to interrupt clinical trials with BAY1161909 in favor of BAY 1217389, which was being developed in parallel. The BAY 1217389 phase I study (NCT02366949) in combination with Paclitaxel was completed in March 2018, but the outcomes are not available yet. 

#### 3.1.2. S 81694

S 81694 inhibits Mps1 with an IC_50_ of 3 nM in vitro, and was first discovered by Nerviano Medical Sciences (NMS-P153), and thereafter acquired and further developed by Servier [23]. S 81694 pre-clinical studies demonstrated that triple-negative breast cancer cell lines were particularly sensitive to S 81694 and, in an MDA-MB-231 xenograft and orthotopic model of tumor regression, the curing of animals and metastasis reduction were observed [24]. A phase I/II trial with S 81694 (NCT03411161) for the treatment of breast cancer was completed in June of 2020, the outcomes of which are not published yet.

#### 3.1.3. BOS 172722 

BOS 172722 is a pyrido[3,4-d]pyrimidine, developed by Boston Pharmaceuticals, and was shown to inhibit Mps1 with an IC_50_ of 11 nM, in vitro [25]. It exhibited modest efficacy as a monotherapy in xenograft studies, but exerted strong synergistic effects in combination with Paclitaxel in triple-negative breast cancer (TNBC) cell lines [26]. A phase I trial (NCT03328494) is currently ongoing with patients with advanced nonhematologic malignancies.

#### 3.1.4. CFI-402257

CFI-402257, a pyrazolo[1,5-a]pyrimidine, is a potent Mps1 inhibitor with an IC_50_ of 1.2 nM in vitro, and it induced tumor regression in murine colon and lung cancer models [27,28]. Currently, two phase I/II trials are recruiting participants: one for the treatment of advanced solid tumors, including breast cancer, as a monotherapy and in combination with Fulvestrant (NCT02792465), and one in combination with Paclitaxel for breast cancer (NCT03568422).

Based on the promising results of in vivo preclinical studies that have demonstrated a synergistic effect between Mps1 inhibitors and taxanes, all small molecules that entered clinical trials were combined with Paclitaxel. However, it is too early to draw a conclusion on the clinical efficacy and safety of these combinations, as only the outcomes of BAY 1161909 have been published to date. Nevertheless, in this trial, the combinational treatment was generally well tolerated, with the most common AEs being gastrointestinal and hematological. Objective responses were observed with BAY 1161909. The rationale behind these combinations is interesting: silencing the SAC by Mps1 inhibition leads to premature mitotic exit with chromosome missegregation, while the affecting of microtubule dynamics by the taxanes further enhances chromosome misalignment and chromosome missegregation, culminating in massive cell death [20]. Inhibition of Mps1 also demonstrated a synergic effect with cisplatin in malignant mesothelioma cisplatin-resistant cell lines in vitro. Therefore, it will be interesting to evaluate the clinical efficacy of Mps1 inhibitors combined with anti-cancer drugs other than taxanes, in particular with platinum-based agents [29]. It should be noted that all clinical trials enrolled patients with solid tumors. Recently, the inhibition of Mps1 was shown to induce apoptosis in multiple myeloma cell lines, but further research is required to evaluate the potential effectiveness of Mps1 inhibitors in hematological tumors [30].

**Table 1:** Mps1 inhibitors in clinical trials ^1^.

Compound	Clinical Trials	Current Status	Conditions	Interventions	Outcomes ^2^	Ref.
BAY 1217389 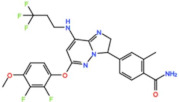	NCT02366949	Phase I Completed	Advanced Solid Malignancies	Combination with Paclitaxel	Not published	-
BAY 1161909 (Empesertib) 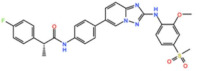	NCT02138812	Phase I Terminated	Advanced Solid Malignancies	Combination with Paclitaxel	Best response was PR	[22]
S 81694 Structure Undisclosed	NCT03411161	Phase I Phase II Completed	Metastatic Breast Cancer, Metastatic Triple-Negative Breast Cancer	Combination with Paclitaxel	Not published	-
BOS 172722 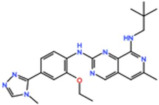	NCT03328494	Phase I Ongoing	Advanced Nonhematologic Malignancies	Combination with Paclitaxel	-	-
CFI-402257 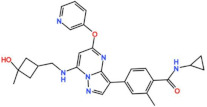	NCT03568422	Phase I Phase II Recruiting	Breast Cancer	Combination with Paclitaxel	-	-
	NCT02792465	Phase I, Recruiting	Breast Cancer, Advanced Solid Malignancies	Monotherapy Combination with Fulvestrant	-	-

^1^ Data collected from clinicaltrials.gov. ^2^ PR, partial response.

### 3.2. Plk1

Plk1 is a cell cycle-regulating serine/threonine kinase implicated in centrosome maturation and separation, mitotic entry, spindle assembly, kinetochore–microtubule attachment, the SAC, DNA damage checkpoint activation, and cytokinesis [31,32,33]. Knockdown or inhibition through small molecules of Plk1 leads to monopolar spindle formation, G2/M arrest, and polyploidy, which ultimately leads to cell death [34,35,36]. Plk1 is overexpressed in several tumors, associated with poor prognosis, and has been associated with resistance to chemotherapeutics such as Doxorubicin, Gemcitabine, and Paclitaxel [37,38]. Taken together, these facts led to the development of several small molecules that target Plk1 for cancer therapy. Nine Plk1 inhibitors have already entered clinical trials: CYC 140, GSK461364, TAK-960, NMS-1286937 (Onvansertib), BI 6727 (Volasertib), BI 2536, Rigosertib, MK-1496, and lipid nanoparticles carrying the siRNA TKM-080301 (Figure 1 and Table 2). Seventy-nine phase I/II clinical trials with solid and hematological tumor patients have been initiated: fifty-two completed, five ongoing, fifteen terminated/withdrawn, and seven are recruiting participants. Additionally, three phase III studies with metastatic pancreatic adenocarcinoma and myelodysplastic syndrome (MDS) patients have been completed, and two studies with acute myeloid leukemia (AML) and MDS patients are ongoing.

#### 3.2.1. BI 2536 and BI6727 (Volasertib)

BI 2536 and Volasertib are two similar molecules developed by Boehringer Ingelheim. BI2536 is a potent ATP-competitive Plk1 inhibitor with an IC_50_ of 0.83 nM in vitro [39]. Several studies have been undertaken with this compound. Overall, the most common AEs were neutropenia and leukopenia. PR were reported in non-small-cell lung cancer patients as monotherapy (2.1%, NCT00376623) and in combination with pemetrexed (5.2%, NCT02211833), and in pancreatic cancer patients (2.3%, NCT00710710) as a monotherapy [40,41,42]. Nevertheless, the greatest efficacy was observed in AML patients (NCT00701766), in which two complete remissions (CRs, 3.7%) and three PRs (5.5%) were reported, and in non-Hodgkin’s lymphoma patients (NCT00243087), also as monotherapy, in which three CR (17.7%) and one PR (5.9%) were achieved [43,44]. However, clinical trials with BI 2536 have been terminated as single agent, and the second-generation inhibitor Volasertib has been chosen for further clinical development.

Volasertib is a dihydropteridinone derivative that inhibits the Plk1 with an IC_50_ of 0.84 nM in vitro [45]. The first-in-human trial of Volasertib was initiated in 2005 against solid tumors as monotherapy (NCT02273388) [46]. The most common AEs were predominately hematological, and the main dose-limiting toxicities (DLT) were thrombocytopenia, neutropenia, and febrile neutropenia. The MTD was established as 400 mg. However, due to overall tolerability, 300 mg was set as the recommended dose for further development [46]. Three PRs (4.6%) were reported in patients with melanoma, ovarian and urothelial cancer, and 40% of patients had the best response of SD [46]. Since then, at least twenty-four more phase I/II trials (twelve completed, six withdrawn, four terminated and two ongoing) have begun against solid and hematological tumors. Several PRs have been reported with Volasertib as a single agent or in combination with other drugs against many solid tumors. In two phase I trials with Volasertib as the single agent against solid tumors, three PRs (3.3%) were reported in patients with melanoma, ureteral cancer (NCT00969553), and gastric cancer (6.7%, NCT01348347) [47,48]. Based on this phase I result of Volasertib as a monotherapy, two phase II trials were conducted in patients with urothelial cancer (NCT01023958) and platinum-resistant/refractory ovarian cancer (NCT01121406). In the study with urothelial cancer patients, seven PRs (14%) were reported and 26% of patients had SD. The safety profile was considered acceptable, but Volasertib demonstrated insufficient antitumor activity for further evaluation as a monotherapy in these patients [49]. Additionally, in the trial with platinum-resistant/refractory ovarian cancer patients, seven PRs (13%) were reported and 44.4% of patients had SD. The expression of Plk1 was evaluated in 47.3% of patients, but the results demonstrated no relationship between Plk1 levels and Volasertib response. In this regard, it was suggested that further clinical development of Volasertib as a single agent in these patients should only be performed after biomarker analysis, in order to select patients with higher chances of response [50]. Another phase II trial was conducted in patients with non-small-cell lung cancer treated with Volasertib as a single agent, in combination with pemetrexed, or pemetrexed alone (NCT00824408). The combinational regimen did not increase toxicity compared to pemetrexed as a single agent. Three PRs (8.1%) were reported with Volasertib as a single agent, and ten (21.3%) in the combinational arm; however, the combination treatment did not demonstrate an efficacy improvement [51]. More objective responses were reported in other studies with solid tumor patients treated with Volasertib in combination with several drugs. In a phase I trial in combination with Nintedanib (NCT01022853), one CR (3.3%) in a breast cancer patient and one PR (3.3%) in a patient with non-small-cell lung cancer were reported [52]. In another phase I study in combination with Afatinib (NCT01206816), two PRs (3.3%) were observed in patients with non-small-cell lung cancer and head and neck cancer of the tongue [53]. Additionally, in a phase I trial, Volasertib was combined with platinum agents (Cisplatin or Carboplatin, NCT00969761); two PRs (6.7%) were reported with Volasertib plus cisplatin in patients with follicular dendritic reticulum cell carcinoma of the palatine tonsil and follicular dendritic reticulum cell retroperitoneal sarcoma, and two additional PRs (6.5%) were also reported with Volasertib plus Carboplatin in patients with non-small-cell lung cancer and hypopharynx carcinoma [54]. Still, the best response was achieved in patients with AML, in which three CRs (15.8%) and another three CRs with incomplete blood count remission (15.8%, CRi) were reported as monotherapy (NCT01662505) [55]. In this trial, the MTD was established as 450 mg, administered intravenously on days 1 and 15 in a 28-day cycle, and grade 3 or higher AEs were mainly hematological (neutropenia and thrombocytopenia). However, the safety profile was considered acceptable and could be managed through the supportive care of granulocyte colony-stimulating factor (G-CSF) [55]. These results prompted the use of Volasertib in a phase II (NCT00804856) and a phase III (NCT01721876) trial against AML, both in combination with cytarabine, which are currently ongoing.

#### 3.2.2. ON 01910.Na (Rigosertib)

Rigosertib inhibits Plk1 with an IC_50_ of 9 nM in vitro [56]. At least thirty-two phase I/II trials have begun (twenty-four completed, one terminated, two withdrawn, three planned/recruiting participants and two ongoing) as monotherapy and in combinational treatments against solid and hematological tumors. Three PRs (9.3%) were reported in Hodgkin lymphoma, thymic cancer and pancreatic ductal adenocarcinoma patients in combination with Gemcitabine (NCT01125891) [57]. These led Rigosertib to a phase III trial against pancreatic adenocarcinoma in combination with Gemcitabine (NCT01360853), which was completed in 2015. In this study the best response was PR in 19% of patients, and SD in 50% of patients; however, the combinational treatment did not demonstrate an improvement in survival compared to Gemcitabine as a single agent [58]. Nevertheless, the phase I/II outcomes in patients with myelodysplastic syndrome (MDS) were the most promising, and Rigosertib was approved for three phase III trials as monotherapy. One is ongoing (NCT02562443), and two have been completed (NCT01241500 and NCT01928537), in which the best overall response was marrow complete remission (mCR) in 20% and 22% of patients, respectively [59,60]. Additionally, in a phase I/II trial in combination with Azacitidine in patients with MDS (NCT01926587), two CRs (12.5%) were reported and the phase II trial is ongoing [61]. Currently, two phase I/II trials with Rigosertib as monotherapy are planned, recruiting participants against recessive dystrophic epidermolysis bullosa (NCT03786237 and NCT04177498), and also a phase I study in patients with non-small-cell lung cancer with Rigosertib in combination with nivolumab is recruiting participants (NCT04263090).

#### 3.2.3. GSK461364

GSK461364 is a thiophene amide that inhibits Plk1 with an IC_50_ of 7 nM in vitro, and it was developed by GlaxoSmithKline [62]. GSK461364 entered a phase I trial for the treatment of advanced solid malignancies as monotherapy (NCT00536835). No objective responses were reported, and only six stable diseases (SD, 15%) were observed in patients with esophageal, endometrial carcinoma, and ovarian cancers, all treated with doses at or above the MTD [63]. The most common AEs were infusion site reactions and phlebitis, and due to the high incidence of venous thromboembolism (20%) no further development is planned.

#### 3.2.4. MK-1496

MK-1496, developed by Merck Sharp & Dohme Corp., has completed a phase I trial as monotherapy for the treatment of advanced solid tumors in 2009 (NCT00880568). The MTD was determined as 80 mg/m^2^, and reversible hematotoxicity was the main side effect [64]. Two PRs (11.8%) were reported in patients with parotid gland carcinoma and small-cell lung cancer, but no further development is planned for MK-1496.

#### 3.2.5. TAK-960

TAK-960, developed by Millennium Pharmaceuticals, inhibits Plk1 with an IC_50_ of 2 nM in vitro [65]. TAK-960 has demonstrated substantial antitumor activity in various human cancer models [66]. A phase I trial was started in 2010 with TAK-960 as monotherapy for the treatment of advanced nonhematologic malignancies (NCT01179399), but has been terminated early due to lack of efficacy, and further development has been halted.

#### 3.2.6. NMS-1286937 (Onvansertib)

Onvansertib, from Nerviano Medical Sciences, is another potent Plk1 inhibitor with an IC_50_ of 2 nM in vitro [67]. In a phase I trial as monotherapy, the best response was SD (26%) in patients with colorectal cancer, pancreatic carcinoma with a K-RAS mutation, head and neck squamous cell carcinoma, and basal cell carcinoma (NCT01014429) [68]. Despite the lack of efficacy as a monotherapy, three phase I/II studies are currently recruiting participants: one for AML in combination with decitabine or cytarabine (NCT03303339), one for metastatic prostate cancer in combination with FOLFIRI (folinic acid, fluorouracil, and irinotecan) and bevacizumab (NCT03829410), and one for metastatic colorectal cancer with a KRAS mutation in combination with abiraterone and prednisone (NCT03414034).

#### 3.2.7. TKM-080301

TKM-080301 is distinctive among the anti-Plk1 since it is a lipid nanoparticle formulation of an siRNA targeting the Plk1 gene transcript. Phase I/II studies on TKM-080301 as monotherapy in patients with advanced hepatocellular carcinomas (HCC, NCT02191878), adrenocortical cancer (ACC, NCT01262235) and other solid tumors (NCT01437007) were completed. TKM-080301 was generally well tolerated, but did not demonstrate clinical antitumor activity against HCC. However, it showed a better response against ACC with a PR (12.5%) reported, and further clinical evaluation is warranted [69,70].

#### 3.2.8. CYC 140

CYC 140, from Cyclacel Pharmaceuticals, inhibits Plk1 with an IC_50_ of 3 nM in vitro, and has demonstrated antitumor activity in human tumor xenografts at non-toxic doses [71]. CYC 140 is the most recent Plk1 inhibitor in clinical trials, and a phase I study using it against hematological malignancies as monotherapy is currently recruiting participants (NCT03884829).

The AEs associated with Plk1 inhibitors were mainly hematological, namely, neutropenia and thrombocytopenia, but the safety profile of most molecules was considered acceptable and manageable. The efficacy outcomes in patients with solid tumors were modest, either as monotherapy or in combinational treatments. Inhibition of Plk1 has been suggested to be more effective in tumors with high levels of Plk1 and mutated p53, thus selection of patients with these tumor characteristics may improve the clinical outcomes of Plk1 inhibitors against solid tumors [74,75]. Interestingly, better responses were achieved in patients with hematological tumors, especially against AML and MDS, with Volasertib and Rigosertib, respectively, representing the most promising molecules among Plk1 inhibitors. Both inhibitors have demonstrated antitumor activity as a monotherapy, which was improved when combined with other chemotherapeutic drugs. Therefore, future efforts should focus on combinational treatments in order to increase efficacy while reducing drug dosage, DLTs, and development of resistance. Additionally, further investigation of biomarkers that predict small molecule efficiency towards inhibiting Plk1 is needed.

**Table 2:** Plk1 inhibitors in clinical trials ^1^.

Compound	Clinical Trials	Current Status	Conditions	Interventions	Outcomes ^2^	Refs.
BI 2536 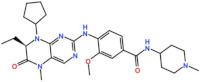	11 Clinical trials	Phase I/II 10 Completed 1 Terminated	Advanced Solid Tumors, Acute Myeloid Leukemia, Non-Hodgkin’s Lymphoma	Monotherapy	Best responses were CR for acute myeloid leukemia and non-Hodgkin’s lymphoma; PR for non-small-cell lung cancer, pancreatic cancer, acute myeloid leukemia, and non-Hodgkin’s lymphoma	[40,41,42,43,44]
				Combination with Pemetrexed and Gemcitabine	Best response was PR for adenocarcinoma and squamous cell carcinoma	
BI 6727 (Volasertib) 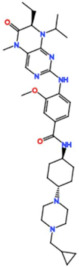	26 Clinical trials	25 Phase I/II 12 Completed 4 Terminated 6 Withdrawn 3 Ongoing	Acute Myeloid Leukemia Pediatric Patients with Advanced Cancers, Myelodysplastic Syndromes, Non-Hodgkin’s Lymphoma, Urothelial, Ovarian, Lung Pancreatic, Colorectal and Prostate Cancer	Monotherapy	Best responses were CR for acute myeloid leukemia; PR for non-small cell lung cancer, melanoma, ovarian cancer, acute myeloid leukemia, gastric cancer, and urothelial cancer	[46,47,48,49,50,51,52,53,54,55]
				Combination with Cytarabine, Pemetrexed, Azacitidine, Afatinib, Decitabine, Daunorubicin, Nintedanib, Mitoxantrone and Itraconazole	Best responses were CR for breast cancer in combination with Nintedanib, PR for non-small-lung cancer in combination with Pemetrexed, Nintedanib, Afatinib, and Carboplatin, PR for head and neck carcinoma in combination with Afatinib, PR for undifferentiated follicular dendritic reticulum cell sarcoma and differentiated follicular dendritic reticulum cell retroperitoneal sarcoma in combination with Cisplatin, PR for differentiated hypopharynx carcinoma in combination with Carboplatin	
		1 Phase III Ongoing	Acute Myeloid Leukemia	Combination with Cytarabine	-	
ON 01910.Na (Rigosertib) 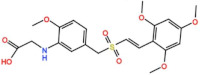	36 Clinical trials	32 Phase I/II 24 Completed 1 Terminated 2 Withdrawn 3 Recruiting/ Planned 2 Ongoing	Refractory Leukemia, Myelodysplastic Syndrome, Squamous Cell Carcinoma Acute/Chronic Myeloid Leukemia, Ovarian and Lung Cancer, Advanced Solid Tumors	Monotherapy	Best response was PR for non-Hodgkin’s lymphoma, thymic cancer, and pancreatic ductal adenocarcinoma	[57,58,59,60,61,72,73]
				Combination with Nivolumab, Cisplatin, Azacitidine, Oxaplatin, Gemcitabine and Irinotecan	Best responses were CR and mCR for myelodysplastic syndrome in combination with Azacitidine	
		4 Phase III 3 Completed 1 Ongoing	Metastatic Pancreatic Adenocarcinoma, Myelodysplastic Syndromes	Monotherapy	Best responses were SD and mCR for myelodysplastic syndromes	
				Combination with Gemcitabine	Best response was PR for pancreatic adenocarcinoma	
GSK461364 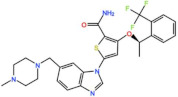	NCT00536835	Phase I Completed	Non-Hodgkin Lymphoma, Advanced Solid Malignancies	Monotherapy	Best response was SD for esophageal and ovarian cancers and endometrial carcinoma	[63]
MK-1496 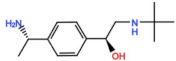	NCT00880568	Phase I Completed	Advanced Solid Tumors	Monotherapy	Best response was PR for parotid gland carcinoma and small cell lung cancer	[64]
TAK-960 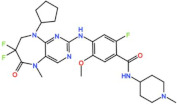	NCT01179399	Phase I Terminated	Advanced Nonhematologic Malignancies	Monotherapy	Discontinued strategically by sponsor due to lack of efficacy	-
NMS-1286937/Onvansertib 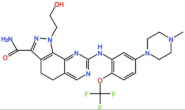	4 Clinical trials	Phase I/II 1 Completed 3 Recruiting	Advanced or Metastatic Solid Tumors	Monotherapy	Best response was SD for colorectal cancer, pancreatic carcinoma with a K-RAS mutation, head and neck squamous cell carcinoma, and basal cell carcinoma	[68]
			Acute Myeloid Leukemia, Metastatic Prostate Cancer, Metastatic Colorectal Cancer with a KRAS mutation	Combination with Decitabine, Cytarabine, Abiraterone, Prednisone, FOLFIRI and Bevacizumab	-	
TKM-080301 (siRNA)	3 Clinical trials	Phase I/II Completed	Hepatocellular Carcinoma, Colorectal, Pancreas, Breast and Ovarian Cancer with Hepatic Metastases, Adrenocortical Carcinoma, Neuroendocrine Tumors	Monotherapy	Best response was PR for adrenocortical carcinoma	[69,70]
CYC 140 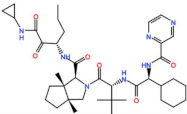	NCT03884829	Phase I Recruiting	Acute Myeloid Leukemia, Myelodysplastic Syndromes, Acute Lymphoblastic Leukemia	Monotherapy	-	-

^1^ Data collected from clinicaltrials.gov. ^2^ CR, complete remission; mCR, marrow complete remission; PR, partial response; SD, stable disease.

## 4. Aurora Kinases

Aurora kinases A, B, and C (AurA, AurB, and AurC) are a family of serine/threonine kinases that play critical functions during mitosis. AurA is implicated in centrosome maturation and separation, cytokinesis, and bipolar spindle assembly, whereas AurB is involved in chromosome condensation and alignment, kinetochore–microtubule attachments, SAC activation, and cytokinesis [76,77]. All Aurora family members are overexpressed in several tumors, and have been related to improving the survival and proliferation of tumor cells [77]. The inhibition of AurA and AurB results in cell death through different mechanisms. AurB inhibition leads to defects in kinetochore–microtubule attachments that override the SAC, resulting in extensive aneuploidy and cell death [78,79]. In contrast, inhibition of AurA provokes a shortened and disorganized microtubule spindle, inducing a transient SAC activation and subsequent mitotic arrest, followed by mitotic exit and apoptosis [80,81]. For these reasons, Aurora kinases were seen as attractive targets for cancer therapy. Therefore, several small molecules with broad-spectrum inhibition activity against Aurora kinases have been developed in pre-clinical studies, known as pan-Aurora inhibitors. Twelve compounds have reached clinical trials: VX-680 (Tozasertib), CYC 116, PHA739358 (Danusertib), SNS-314 Mesylate, PF-03814735, AS703569 (Cenisertib), TAK-901, ABT-348 (Ilorasertib), AMG-900, GSK1070916 (NIM-900), AT9283, and BI-847325 (Figure 1 and Table 3).

### 4.1. Pan-Aurora Inhibitors

#### 4.1.1. ABT-348 (Ilorasertib)

Ilorasertib was reported to inhibit AurA with an IC_50_ of 120 nM, AurB with an IC_50_ of 7 nM, and AurC with an IC_50_ of 1nM, in vitro, and is also able to inhibit the VEGF [82]. At least four phase I/II trials have been initiated with Ilorasertib against solid and hematological tumors. A phase I trial was conducted with patients with solid tumors in which Ilorasertib was administered as single agent, or in combination with Docetaxel or Carboplatin (NCT01110486). However, only the outcomes as monotherapy were published. In total, 10% of patients experienced a total of ten DLTs, and the study was terminated by strategic decision of the sponsor without establishment of MTD. Nevertheless, two PRs (2.4%) were reported in patients with basal cell carcinoma and adenocarcinoma [83]. In a phase I trial in patients with hematological tumors, Ilorasertib was administered in combination with Azacitidine (NCT01110473). The DLTs observed were pancreatitis, acute kidney injury, and hypertension. Among the fifty-two patients, twelve had SD as the best response, and three objective responses (CR, CRi, and PR) were reported in patients with AML [84]. Currently, a phase II trial is ongoing with Ilorasertib as a single agent (NCT02478320).

#### 4.1.2. AS703569/MSC1992371A (Cenisertib)

Cenisertib displays a broad inhibitory activity against a number of kinases, including AurA and B [85]. A phase I trial using it as monotherapy was conducted in patients with solid tumors (NCT00391521). Neutropenia, febrile neutropenia, and thrombocytopenia were the most common DLTs observed, and no objective responses were observed [86]. In another phase I trial with patients with solid tumors, Cenisertib was administered in combination with Gemcitabine (NCT01097512). The MTD was 37 mg/m^2^ of Cenisertib with the standard 1000 mg/m^2^ of Gemcitabine, and neutropenia was the main DLT reported. The best response observed was two PRs (3%) in patients with non-small-cell lung cancer and hepatocellular carcinoma [87]. A phase I trial was also conducted with patients with hematological malignancies with Cenisertib as single agent in two schedules (NCT01080664). In the first schedule, Cenisertib was given on days 1–3 and 8–10, and in the second schedule on days 1–6, both in a 21-day cycle. The MTDs were established as 37 mg/m^2^ and 28 mg/m^2^ for the first and second schedules, respectively. The DLTs reported were severe neutropenia with infection and sepsis, mucositis, and diarrhea. Overall, two CRs (2.7%) were observed in patients with AML, and a CRi (1.3%) was reported in a patient with acute lymphoid leukemia with Philadelphia chromosome [88].

#### 4.1.3. VX-680 (Tozasertib)

Tozasertib, also known as VX-680 or MK-0457, was developed by Merck Sharp & Dohme Corp., and was shown to inhibit AurA with an IC_50_ of 0.6 nM, AurB with an IC_50_ of 18 nM, and AurC with an IC_50_ of 4.6 nM, in vitro [89]. Tozasertib has entered five trials as monotherapy against solid and hematological tumors, and in combination with Dasatinib in patients with chronic myeloid leukemia and Philadelphia chromosome-positive acute lymphoblastic leukemia (NCT00500006). In the first-in-human study in patients with solid tumors (NCT02532868), the MTD was identified as 64 mg/m^2^, and Tozasertib was generally well tolerated, with neutropenia and a herpes zoster reactivation being the DLTs observed. No objective responses were reported, and twelve patients (44.4%) had SD as best response [90]. The best response to Tozasertib treatment was observed in a trial with patients with leukemia (NCT00111683), in which a CR (1.3%) was reported in a patient with chronic myeloid leukemia [91].

#### 4.1.4. BI-847325

BI-847325, from Boehringer Ingelheim, is a potent inhibitor of all Aurora family members as well as MEK1/2 kinases [92]. A phase I study with BI-847325 as the single agent in patients with solid tumors was initiated in 2011 (NCT01324830). The DLTs observed were primarily hematological (neutropenia, febrile neutropenia, and thrombocytopenia) and gastrointestinal (vomiting and diarrhea). In total, 45% of patients had SD as the best response, and one PR (1.4%) was reported in a patient with squamous cell carcinoma of the esophagus [93].

#### 4.1.5. AT9283

AT9283, from Astex Pharmaceuticals, inhibits AurA and AurB with an IC_50_ of 3 nM in vitro. Additionally, AT9283 was also found to inhibit other kinases including JAK2, Flt3, and Abl (T315I) [94]. At least five trials have been completed with AT9283 as monotherapy. A phase I trial in patients with solid tumors or non-Hodgkin’s lymphoma was started in 2007 (NCT00443976). The MTD was determined to be 47 mg/m^2^/day, and febrile neutropenia and wound infection were the DLTs observed. Four patients (12.5%) achieved SD as best response and one PR (3.1%) was reported in a patient with squamous cell carcinoma of the anal canal [95]. In a phase II trial with patients with leukemia or myelofibrosis (NCT00522990), the MTD was established as 324 mg/m^2^/72h, and tolerability was strongly dose-dependent. No CR or PR were observed [96]. A phase I trial was conducted in children and adolescents with solid tumors (NCT00985868). The MTD was 18.5 mg/m^2^/day, and the most common DLTs observed were neutropenia and febrile neutropenia. The best response reported was a PR (4.3%) in a patient with central nervous system-primitive neuroectodermal tumor [97].

#### 4.1.6. AMG-900

AMG-900, developed by Amgen, inhibits Aurora A, B, and C in vitro, with IC_50_ values of 5 nM, 4 nM, and 1 nM, respectively [98]. The first-in-human trial of AMG-900 was conducted in patients with solid tumors (NCT00858377). The MTD was 25 mg/day, and neutropenia was the most common DLT observed. Consequently, G-CSF support was included in treatment regimen, and the MTD was established as a higher dose of 40 mg/day. In total, 56% of the patients had SD and one PR (2.4%) was reported in a patient with clear-cell endometrial cancer [99]. In a phase I trial against AML (NCT01380756) with AMG-900 as monotherapy, the most common AEs were nausea, diarrhea, and febrile neutropenia. Three patients had a best response of CRi (9%) and no other responses were observed [100].

#### 4.1.7. PHA739358 (Danusertib)

Danusertib is a pyrrolo-pyrazole developed by Nerviano Medical Sciences, and inhibits AurA with an IC_50_ of 13 nM, AurB with an IC_50_ of 79 nM, and AurC with an IC_50_ of 61 nM, in vitro [101]. A phase II trial in patients with multiple myeloma was terminated earlier due to low recruitment rate (NCT00872300). Another phase II trial with Danusertib as single agent against metastatic hormone-refractory prostate cancer was completed (NCT00766324). Danusertib was generally well tolerated with neutropenia and fatigue being the most common AEs observed. No objective responses were observed, and 21 (25.9%) patients achieved SD as the best response [102].

#### 4.1.8. SNS-314 Mesylate

SNS-314 Mesylate, from Sunesis Pharmaceuticals, inhibits AurA with an IC_50_ of 9 nM, AurB with an IC_50_ of 31 nM, and AurC with an IC_50_ of 3 nM, in vitro [103]. SNS-314 Mesylate entered a phase I trial (NCT00519662) in patients with solid tumors as a single agent. It was generally well tolerated, with nausea, fatigue, vomiting, constipation, and pain being the most AEs commonly observed. In total, 18.8% of patients had SD as the best response, and no objective responses were reported [104].

#### 4.1.9. TAK-901

TAK-901 was developed by Millennium Pharmaceuticals (Takeda) and inhibits AurA and B in vitro, with IC_50_ values of 21 nM and 15 nM, respectively [105]. TAK-901 entered two phase I trials for solid and hematological malignancies (NCT00935844 and NCT00807677). Both trials were completed but no data are available yet.

#### 4.1.10. CYC116

CYC116, from Cyclacel Pharmaceuticals, inhibits AurA with a Ki of 8 nM and AurB with a Ki of 9.2 nM, and showed antitumor activity in vivo [106]. CYC116 entered a phase I trial in patients with solid tumors as monotherapy (NCT00560716); however, the study was terminated early by sponsor decision.

#### 4.1.11. GSK1070916 (NIM-900)

GSK1070916, also known as NIM-900, is a potent Aurora B and C inhibitor, with IC_50_ values of 5 nM and 6.5 nM, respectively, in vitro [107]. A phase I trial in patients with advanced solid tumors was completed in 2013 with GSK1070916 as monotherapy (NCT01118611), but the outcomes are not published yet.

#### 4.1.12. PF-03814735

PF-03814735, developed by Pfizer, is a potent Aurora A and B inhibitor with IC_50_ values of 0.8 nM and 5 nM, respectively, in vitro [108]. PF-03814735 entered a phase I trial against solid tumors as a single agent (NCT00424632). The DLTs observed were neutropenia, febrile neutropenia, increase in aspartate amino transferase, and left ventricular dysfunction. No objective responses were reported, with 35.5% of patients achieving SD as the best response [109].

**Table 3:** Pan-Aurora inhibitors in clinical trials ^1^.

Compound	Clinical Trials	Current Status	Conditions	Interventions	Outcomes ^2^	Refs.
ABT-348 (Ilorasertib) 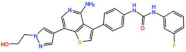	4 Clinical trials	Phase I/II 3 Completed 1 Ongoing	Solid Tumors, Advanced Hematological Malignancies	Monotherapy	Best response was PR for basal cell carcinoma, and adenocarcinoma	[83,84]
				Combination with Carboplatin, Docetaxel and Azacitidine	Best responses were CR, PR, and CRi for acute myeloid leukemia in combination with Azacitidine	
AS703569/MSC1992371A (Cenisertib) 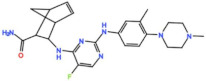	3 Clinical trials	Phase I/II 2 Completed 1 Terminated	Solid Tumors, Advanced Malignancies	Monotherapy	Best response was CR for acute myeloid leukemia and CRi for acute lymphoid leukemia with Philadelphia chromosome	[86,87,88]
				Combination with Gemcitabine	Best response was PR for non-small-cell lung cancer and hepatocellular carcinoma	
VX-680/MK-0457 (Tozasertib) 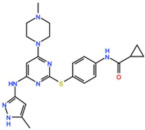	6 Clinical trials	Phase I/II 1 Completed 5 Terminated	Chronic Myeloid Leukemia, Philadelphia Chromosome-Positive, Acute Lymphoblastic Leukemia, Non-Small-Cell Lung Carcinoma, Advanced Solid Tumors	Monotherapy Combination with Dasatinib	Best response was one CR for chronic myeloid leukemia with BCR–ABL T315I mutation phenotype as monotherapy	[90,91,110]
BI-847325 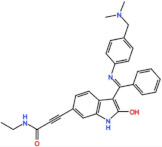	NCT01324830	Phase I Completed	Solid Tumors	Monotherapy	Best response was PR for esophageal cancer	[93]
AT9283 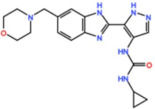	5 Clinical trials	Phase I/II 4 Completed 1 Terminated	Multiple Myeloma, Acute Leukemia, Myelofibrosis, Advanced or metastatic solid tumors, Non-Hodgkin’s lymphoma, Solid Tumors	Monotherapy	Best response was PR for nervous system primitive neuroectodermal tumor and squamous cell carcinoma	[95,96,97]
AMG-900 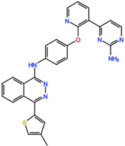	NCT00858377	Phase I Completed	Advanced Solid Tumors	Monotherapy	Best response was PR for clear-cell endometrial cancer	[99]
	NCT01380756	Phase I Completed	Acute Myeloid Leukemia	Monotherapy	Best response was CRi	[100]
PHA739358, (Danusertib) 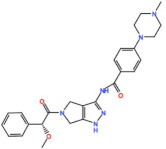	NCT00766324	Phase II Completed	Metastatic Hormone Refractory Prostate Cancer	Monotherapy	The best response was SD	[102]
	NCT00872300	Phase II Terminated	Multiple Myeloma		-	-
SNS-314 Mesylate 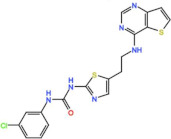	NCT00519662	Phase I Completed	Advanced Solid Tumors	Monotherapy	Best response was SD	[104]
TAK-901 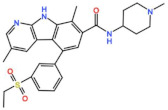	NCT00935844	Phase I Completed	Advanced Solid Tumors Lymphoma	Monotherapy	Not published	-
	NCT00807677	Phase I Completed	Advanced Hematologic Malignancies			
CYC116 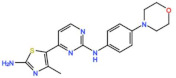	NCT00560716	Phase I Terminated	Advanced Solid Tumors	Monotherapy	-	-
GSK1070916/NMI-900 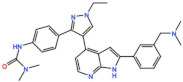	NCT01118611	Phase I Completed	Advanced Solid Tumors	Monotherapy	Not published	-
PF-03814735 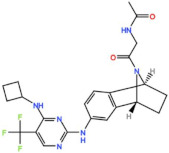	NCT00424632	Phase I Completed	Advanced Solid Tumors	Monotherapy	Best response was SD for non-small-cell lung cancer, melanoma, renal cell carcinoma, and neuroendocrine tumor	[109]

^1^ Data collected from clinicaltrials.gov. ^2^ CR, complete remission; Cri, complete remission with incomplete blood count remission; PR, partial response; SD, stable disease.

### 4.2. Aurora B inhibitors

In addition to inhibitors with a broad-spectrum for Aurora kinases, several small molecules have been developed with selectivity to each Aurora family member. Four AurB-specific inhibitors (Chiauranib, AZD1152 or Barasertib, BI-811283, and BI-831266) have entered clinical trials for the treatment of solid and hematological tumors (Figure 1 and Table 4).

#### 4.2.1. AZD1152 (Barasertib)

Barasertib, from AstraZeneca, is a potent AurB inhibitor with an IC_50_ of 0.37 nM in vitro [111]. Barasertib entered at least ten clinical trials for the treatment of solid and hematological tumors. A phase I trial as single agent was conducted in patients with solid tumors (NCT00338182). Fatigue, neutropenia, and nausea were the most common AEs, and neutropenia was the main DLT observed. No objective responses were reported, and 26.5% of patients had SD as the best response [112]. In another phase I trial with patients with solid tumors (NCT00497731), the outcomes were similar to the previous study, in which neutropenia was the main DLT observed and the best response reported was SD in 25.5% of patients [113]. Despite the lack of efficacy in solid tumors, Barasertib demonstrated better antitumor activity in patients with AML. In a phase I/II trial (NCT00497991) using it as a single agent, the MTD was established as 1200 mg, with febrile neutropenia and stomatitis/mucosal inflammation being the most common AEs observed. Sixteen (25%) objective responses were observed, including three CRs, six CRi, and seven PRs [114]. Furthermore, additional responses (CR, CRi, or PR) were reported in other trials (NCT00530699, NCT00952588, and NCT01019161) with Barasertib as a single agent in patients with AML [115,116,117]. Another phase I trial was conducted in AML patients in combination with low doses of Cytarabine (NCT00926731). The MTD was established as 1000 mg of Barasertib and 400 mg of Cytarabine, with the most common AEs being febrile neutropenia, nausea, diarrhea, peripheral edema, and stomatitis. Ten patients (45%) had a response to treatment, including six CR, two CRi, and two PRs [118]. A phase I/II trial with AML patients is currently recruiting participants with Barasertib as a single agent, or in combination with Venetoclax and Azacitidine (NCT03217838). A phase II trial was also conducted with patients with diffuse large B-cell lymphoma (NCT01354392) with Barasertib as a single agent. Patients received up to six cycles of 800 mg of Barasertib starting on day 1 of the 21-day cycle, and G-CSF support was added to the regimen if grade 3 or higher neutropenia occurred. The most common AEs were neutropenia, nausea, diarrhea, anemia, fatigue, and mucositis. In total, 33% of patients had SD and three PRs (20%) were reported [119].

#### 4.2.2. BI-831266

BI-831266 inhibits AurB with an IC_50_ of 42 nM in vitro and has demonstrated antitumor activity in murine xenograft tumor models [120]. A phase I trial as monotherapy was conducted with patients with solid tumors (NCT00756223). The most common AEs were fatigue, neutropenia, and alopecia, with febrile neutropenia being the only DLT observed. In total, 16% of patients had SD, and one PR (4%) was reported in a patient with cervical cancer [121].

#### 4.2.3. BI-811283

BI-811283 is another small molecule developed by Boehringer Ingelheim that inhibits AurB with an IC_50_ of 9 nM in vitro [122]. A phase I study with patients with solid tumors was completed with BI-811283 as a single agent (NCT00701324). The DLTs observed were mainly hematological, including neutropenia and thrombocytopenia. No objective responses were reported, and 37% of patients had SD as the best response [123]. Better results were observed in a phase II trial with BI-811283 in combination with a low dose of cytarabine in AML patients (NCT00632749). MTD was established as 100 mg of BI-811283, with anemia, nausea, pyrexia, and febrile neutropenia being the most common AEs observed. In total, 14% of patients showed treatment responses, including seven CR, one CRi, and one PR [124].

#### 4.2.4. Chiauranib

Chiauranib, developed by Chipscreen Biosciences, is a potent AurB inhibitor with an IC_50_ of 9 nM in vitro. Chiauranib also exerts an inhibitory activity against VEGF receptors and the colony-stimulating receptor 1 (CSF-1R), and is the most recent AurB inhibitor tested in trials [125]. A phase I trial monotherapy was conducted with patients with solid tumors (NCT02122809). Chiauranib was generally well tolerated, with fatigue, proteinuria, hematuria, hypothyroidism, hypertriglyceridemia, and hypertension being the most common AEs observed. No objective responses were observed, with 66.7% of patients achieving SD as best response [126]. Currently, a phase II trial is ongoing with ovarian cancer patients (NCT03901118), and another three phase I/II studies are planned, recruiting participants with hepatocellular carcinoma (NCT03245190), non-Hodgkin’s lymphoma (NCT03974243), and small-cell lung cancer (NCT03216343).

**Table 4:** Aurora B inhibitors in clinical trials ^1^.

Compound	Clinical Trials	Current Status	Conditions	Interventions	Outcomes ^2^	Refs.
AZD1152 (Barasertib) 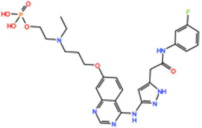	10 Clinical trials	Phase I/II 8 Completed 2 Terminated	Lymphoma, Advanced Solid Tumors, Acute Myeloid Leukemia	Monotherapy	Best response was CR for acute myeloid leukemia, and PR for lymphoma and acute myeloid leukemia	[112,113,114,115,116,117,118,119]
				Combination with low dose of Cytarabine, Venetoclax, Azacitidine	Best response was CR and PR for acute myeloid leukemia in combination with low dose of Cytarabine	
BI-831266 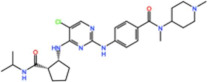	NCT00756223	Phase I Completed	Advanced Solid Tumors	Monotherapy	Best response was PR for cervical cancer	[121]
BI-811283 (Structure Undisclosed)	NCT00701324	Phase I Completed	Solid Tumors	Monotherapy	Best response was SD	[123]
	NCT00632749	Phase II Completed	Acute Myeloid Leukemia	Combination with Cytarabine	Best responses were CR, PR, and CRi	[124]
Chiauranib 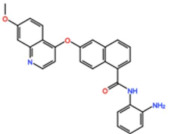	7 Clinical trials	Phase I/II 2 Completed 1 Terminated 3 Recruiting/ Planned 1 Ongoing	Ovarian Cancer, Non-Hodgkin’s Lymphoma, Hepatocellular Carcinoma, Small Cell Lung Cancer, Other Advanced Solid Tumors	Monotherapy	Best response was SD	[126]

^1^ Data collected from clinicaltrials.gov. ^2^ CR, complete remission; Cri, complete remission with incomplete blood count remission; PR, partial response; SD, stable disease.

### 4.3. Aurora A inhibitors

Inhibitors with selectivity to AurA were also developed. In fact, more AurA inhibitors have reached clinical trials than AurB inhibitors, including MLN8237 (Alisertib), LY3295668 (Erbumine), TAS-119, ENMD-2076, MK-5108/VX-689, KW-2449, and MLN8054. At least seventy-three trials involving these compounds have been initiated for the treatment of solid and hematological tumors (Figure 1 and Table 5).

#### 4.3.1. MLN8237 (Alisertib)

Alisertib is a potent oral AurA inhibitor with an IC_50_ of 1.2 nM in vitro [127]. Alisertib has been extensively tested in clinical trials, with at least fifty-four trials initiated as monotherapy, and in several combinational treatments against solid and hematological tumors.

The first-in-human trial was conducted in patients with solid tumors with Alisertib as a single agent (NCT00500903). Patients were administered orally with Alisertib for 7, 14, or 21 consecutive days, followed by a 14-day recovery period. MTD was established as 50 mg twice a day for 7 consecutive days, and the main DLTs observed were neutropenia and thrombocytopenia. Alisertib was generally well tolerated, with 38% of patients achieving SD as the best response, and one PR (1.1%) was reported in a patient with platinum- and radiation-refractory ovarian cancer lasting for more than one year [128]. These results prompted the undertaking of more trials with Alisertib as a single agent in patients with solid tumors. Objective responses were reported. In a phase II trial in patients with ovarian, fallopian tube, or peritoneal carcinoma (NCT00853307), two (6%) PRs were reported in patients with ovarian carcinoma [129]. In two phase II trials in patients with metastatic sarcoma (NCT01653028) and metastatic castrate-resistant and neuroendocrine prostate cancer (NCT01799278), two PRs in each study (2.7% and 3.3%, respectively) were reported [130,131]. Additionally, several studies tested the combination of Alisertib with taxane drugs (Paclitaxel and Docetaxel) in patients with solid tumors. A phase I/II trial was conducted in patients with breast or ovarian carcinoma, who received Alisertib in combination with Paclitaxel or Paclitaxel alone (NCT01091428). The MTD was established as 10 mg of Alisertib twice a day plus 80 mg/m^2^ of Paclitaxel. Febrile neutropenia, neutropenia, stomatitis, and diarrhea were the DLTs observed, although this combination has demonstrated a manageable safety profile. Seven CRs (11%) and 23 PRs (37%) were reported in the combinational treatment schedule, representing a 10% improvement in the ORR, in comparison to Paclitaxel as single agent (30% vs. 20%) [132]. More objective responses with Alisertib in combination with Paclitaxel were also reported in other solid tumors. For instance, in a phase II trial for the treatment of small-cell lung cancer (NCT02038647), nineteen PRs (21%) and one CR were reported (1.1%), and in a phase II with urothelial cancer patients (NCT02109328), two PRs (9.1%) were observed [133,134]. The efficacy outcomes of a phase I trial which combined Alisertib with Docetaxel in solid tumor patients (NCT01094288) were similar to those for the combination with Paclitaxel, in which seven PRs (25%) were reported in patients with angiosarcoma and castration-resistant prostate cancer, and a CR (3,6%) was observed in a patient with bladder cancer [135]. Furthermore, additional PRs in patients with solid tumors were reported with Alisertib in combination with other drugs, such as Pazopanib against breast cancer and mesothelioma (7%, NCT01639911), Irinotecan and Temozolomide against neuroblastoma (12.5%, NCT01601535), Oxaliplatin, Leucovorin, and Fluorouracil against colorectal cancer (8.3%, NCT02319018), and Fulvestrant against lobular ER+/PR+/HER2-breast cancer (22.2%, NCT02219789) [136,137,138,139]. Currently, several phase I/II trials are ongoing, namely, with lung cancer and mesothelioma patients treated with Alisertib as single agent (NCT02293005), with solid tumor patients in combination with Gemcitabine (NCT01924260), with breast cancer patients in combination with Paclitaxel (NCT02187991), and with solid tumor and breast cancer patients in combination with MLN0128 (NCT02719691). Additionally, other trials are recruiting participants for the treatment of head and neck squamous cell carcinoma and malignant solid neoplasm with Alisertib in combination with Pembrolizumab (NCT04555837), and non-small-cell lung cancer (NCT04479306) and EGFR-mutant lung cancer (NCT04085315) with Alisertib in combination with Pembrolizumab.

The clinical trial results of Alisertib against hematological tumors were also very promising. A phase I monotherapy study was conducted (NCT00697346). The recommended phase II dose was 50 mg twice a day for 7 days, followed by a recovery period of 14 days, in a 21-day cycle. The DLTs observed were mainly hematological, including neutropenia, febrile neutropenia, and thrombocytopenia, with 9% of patients who had their treatment discontinued due to AEs. However, Alisertib was generally well tolerated at the recommended phase II dose. In total, 27.6% of patients had SD as the best response, and six PRs (12.8%) were reported in patients with follicular lymphoma, multiple myeloma, peripheral T-cell lymphoma, and diffuse large B-cell lymphoma [140]. These results encouraged the use of Alisertib in a phase II study in patients with T-cell lymphoma as monotherapy (NCT01466881). Patients received the recommended phase II dose established in the previous study. The most common grade 3 or higher AEs were neutropenia, anemia, and thrombocytopenia. In total, 18.9% of patients had SD, and the best responses observed were two CRs (5.4%) and seven PRs (18.9%) [141]. Based on the efficacy results observed in this study, a phase III trial (NCT01482962) was conducted in patients with peripheral T-cell lymphoma, in which Alisertib efficacy as a single agent was evaluated and compared to a comparator (Pralatrexate, Gemcitabine, or Romidepsin). Despite the ORR with Alisertib being 33% (18 CRs and 16 PRs), it was not statistically significantly superior to the comparator arm [142]. However, objective responses were reported in other hematological tumors too. In a phase II trial with non-Hodgkin lymphoma patients treated with Alisertib as a single agent (NCT00807495), 10% of patients had CR and 17% had PR [143]. In a phase I/II trial conducted in patients with non-Hodgkin lymphomas (NCT01397825), treated with Alisertib in combination with Rituximab or with Rituximab plus Vincristine, 38% ORR was observed, including seven CRs and seven PRs [144]. Additionally, six PRs (23%) were observed in a phase I/II trial with multiple myeloma patients (NCT01034553) treated with Alisertib in combination with Bortezomib [145]. In a phase II trial in patients with AML (NCT02560025), treated with Alisertib combined with induction chemotherapy (Daunorubicin or Idarubicin plus Cytarabine), 51% of patients had CR and five CRi (13%) were also reported [146]. Currently, a phase I trial is ongoing in patients with non-Hodgkin lymphomas, with Alisertib in combination with Bortezomib and Rituximab (NCT01695941).

Alisertib was also evaluated in children with solid and hematological tumors. A phase I trial was conducted in pediatric patients with solid tumors, with Alisertib as single agent (NCT02444884). Neutropenia was the most common DLT observed, and the MTD was established as 80 mg/m^2^ once daily for 7 days followed by 2 weeks rest in a 21-day cycle. In total, 18% of patients had SD and one PR (3%) was reported in a patient with hepatoblastoma [147]. A phase II trial combined Alisertib with Paclitaxel in patients with solid tumors or leukemia (NCT01154816). The most common AEs were neutropenia, anemia, leukopenia, and thrombocytopenia. One CR (0.7%) and two PRs (1.5%) were observed in patients with neuroblastoma and another patient with Wilms tumor also had a CR [148]. Currently, a phase II trial using Alisertib as a single agent against rhabdoid tumor is recruiting participants (NCT02114229).

#### 4.3.2. ENMD-2076

ENMD-2076 is an orally administered AurA inhibitor with an IC_50_ of 14 nM, and is also capable of inhibiting multiple tyrosine kinases in vitro [149]. ENMD-2076 entered eight phase I/II trials for the treatment of solid and hematological tumors, all as monotherapy. The first-in-human trial was conducted in patients with solid tumors (NCT00658671). MTD was established as 160 mg/m^2^, and the DLTs observed were neutropenia and hypertension. In total, 85% of patients achieved SD as the best response, and two PRs (3%) were reported in patients with platinum-refractory/resistant ovarian cancer [150]. These results prompted the conduction of two phase II studies with ENMD-2076 in patients with ovarian cancer. Overall, ENMD-2076 was well tolerated, with hypertension and diarrhea being the most common AEs in both studies. In the first trial (NCT01104675), with patients with platinum-refractory/resistant ovarian cancer, the majority of patients (52%) had progressive diseases, although five PRs (8%) were reported [151], whereas in the other study (NCT01914510), with patients with clear-cell ovarian cancer, 55% of patients achieved SD and two PRs (7.9%) were observed [152]. Additional objective responses were also observed in other tumors. A phase II trial was conducted in patients with soft tissue sarcoma (NCT01719744), in which 35% of patients had SD and two PRs (9%) were reported in patients with angiosarcoma and undifferentiated pleomorphic sarcoma [153]. In a phase II trial with triple-negative breast cancer patients (NCT01639248), 38.9% of patients had SD and two PRs (5.2%) were reported [154]. Furthermore, in a phase II trial with fibrolamellar carcinoma patients (NCT02234986), one PR (3%) was reported and 57% of patients had SD [155]. Additionally, ENMD-2076 entered two trials against hematological tumors. A phase I trial with patients with AML or chronic myelomonocytic leukemia was completed in 2011 (NCT00904787). MTD was established as 225 mg once a day, with typhilis, fatigue, syncope, and QTc prolongation being the DLTs observed. The best response was observed in three patients who had CRi (19%) [156].

#### 4.3.3. LY3295668 (Erbumine)

LY3295668 is a potent AurA inhibitor with an IC_50_ of 1.12 nM in vitro, and it has demonstrated antitumor activity in xenograft cancer models [80]. LY3295668 is the most recently tested inhibitor in clinical trials, with two trials completed. The first-in-human trial with LY3295668 as monotherapy was conducted in patients with solid tumors (NCT03092934). MTD was 25 mg twice a day, with diarrhea, corneal deposits, and mucositis being the DLTs observed. The best response was SD achieved in 69% of patients [157]. The other phase I/II study, with metastatic breast cancer patients, was completed in 2020; the results are yet to be announced (NCT03955939). Currently, a phase I trial is ongoing for the treatment of small-cell lung cancer (NCT03898791) with LY3295668 as monotherapy, and another phase I study with LY3295668 in combination with Topotecan and Cyclophosphamide in patients with neuroblastoma is recruiting participants (NCT04106219).

#### 4.3.4. MLN8054

MLN8054, from Millennium Pharmaceuticals, inhibits AurA with an IC_50_ of 4 nM in vitro [158]. The first-in-human trial was conducted with patients with solid tumors (NCT00249301), in which MLN8054 was given orally for 7, 14, or 21 days, followed by a 14-day recovery period. The MTD was 60 mg, with the most common DLT observed being somnolence. No objective responses were observed, and 15% of patients had SD as the best response [159]. In another phase I trial with patients with solid tumors (NCT00652158), the efficacy outcomes were similar, in which the best response observed was SD [160].

#### 4.3.5. MK-5108 (VX-689)

MK-5108, from Merck Sharp & Dohme, is a highly potent AurA inhibitor with an IC_50_ of 0.064 nM in vitro [161]. A phase I trial was conducted in patients with solid tumors, in which MK-5108 was given as single agent and in combination with Docetaxel (NCT00543387). Its MTD as a single agent was not established because no patients experienced a DLT, whereas the MTD of the combinational treatment was 300 mg/day. Overall, the drug-related AEs were mainly blood and lymphatic system disorders. No objective responses were reported with MK-5108 as a single agent. However, a PR (5.9%) was observed in a patient who received 450mg/day and a standard dose of Docetaxel [162].

#### 4.3.6. TAS-119

TAS-119, developed by Taiho Oncology, is a potent AurA inhibitor with an IC_50_ of 1 nM in vitro [163]. The first-in-human phase I study with TAS-119 as monotherapy was conducted in patients with solid tumors (NCT02448589). The MTD was established as 250 mg twice a day, with fatigue, pain, and diarrhea being the most common AEs observed. No objective responses were observed, and 35% of patients had SD as the best response [164]. In a phase I trial for the treatment of solid tumors, TAS-119 was given in combination with Paclitaxel (NCT02134067). The MTD was established as 80 mg/m^2^ of Paclitaxel with 75 mg of TAS-119 twice a day. Neutropenia and elevated AST were the DLTs observed. In total, 45% of patients had SD, and four PRs (15.4%) were reported in patients with ovarian/fallopian tube cancers [165].

#### 4.3.7. KW-2449

KW-2449, developed by Kyowa Kirin Pharmaceutical Development, is a multikinase inhibitor, active against AurA with an IC_50_ of 48 nM in vitro [166]. KW-2449 entered two phase I trials with patients with hematological tumors. However, both were terminated due to a suboptimal dosing schedule and failure to identify a tolerable dose that had potential efficacy (NCT00346632 and NCT00779480).

**Table 5:** Aurora A kinase-specific inhibitors in clinical trials ^1^.

Compound	Clinical Trials	Current Status	Conditions	Interventions	Outcomes ^2^	Refs.
MLN8237 (Alisertib) 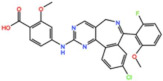	53 Clinical trials	Phase I/II 38 completed 7 terminated 4 ongoing 4 recruiting	Advanced Hematological Malignancies, Advanced Solid Tumors, Lymphoma, Prostate Cancer, Ovarian, Fallopian Tube and Peritoneal Carcinoma, Leiomyosarcoma, Neuroblastoma, Acute Myeloid Leukemia, Mantle Cell Lymphoma, Burkitt’s Lymphoma, Breast Carcinoma, Glioma, Non-Hodgkin’s Lymphoma, Bladder Cancer, Non-Small-Cell Lung Cancer, Mesothelioma, Small-Cell Lung Cancer Adenocarcinoma, Melanoma, Head and Neck Squamous Cell Carcinoma, EGFR-mutant Lung Cancer, Rhabdoid Tumor	Monotherapy	Best responses were CR for neuroblastoma, Wilms tumor, non-Hodgkin’s lymphoma, and acute myeloid leukemia; PR for lymphoma, multiple myeloma, angiosarcoma, ovarian cancer, prostate cancer, hepatoblastoma, neuroblastoma, non-Hodgkin’s lymphoma, and acute myeloid leukemia	[128,129,130,131,132,133,134,135,136,137,138, 139,140,141,143,144,144,145,146,147,148]
				Combination with Pazopanib, Bortezomib, Docetaxel, Irinotecan, Temozolomide, Daunorubicin, Idarubicin, Cytarabine, Rituximab, Vincristine, Paclitaxel, Oxaliplatin, Leucovorin, Fluorouracil, Esomeprazole, Rifampin, Fulvestrant, Itraconazole, Idarubicin, Cytarabine, Vorinostat, MLN0128, Abiraterone Acetate, Prednisone, Erlotinib, Gemcitabine, Pembrolizumab, Osimertinib, Romidepsin, Bortezomib	Best responses were CR for multiple myeloma in combination with Bortezomib, ovarian cancer in combination with Paclitaxel acute myeloid leukemia in combination with Daunorubicin, Idarubicin, and Cytarabine, refractory aggressive B-cell lymphoma in combination with Rituximab and Vincristine, breast cancer in combination with Paclitaxel, small-cell lung cancer in combination with Paclitaxel; PR for urothelial cancer in combination with Paclitaxel, breast cancer in combination with Pazopanib, Fulvestrant and Paclitaxel, mesothelioma in combination with Pazopanib, multiple myeloma in combination with Bortezomib, angiosarcoma in combination with Docetaxel, castration-resistant prostate cancer in combination with Docetaxel, neuroblastoma in combination with Temozolomide, acute myeloid leukemia in combination with Daunorubicin, Idarubicin, and Cytarabine, refractory aggressive B-cell lymphoma in combination with Rituximab and Vincristine, ovarian cancer in combination with Paclitaxel, colon cancer in combination with Oxaliplatin, Leucovorin, and Fluorouracil, small-cell lung cancer in combination with Paclitaxel	
MLN8237 (Alisertib) 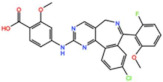	NCT01482962	Phase III Completed	Relapsed/Refractory Peripheral T-Cell Lymphoma	Monotherapy	Best responses were CR and PR	[142]
ENMD-2076 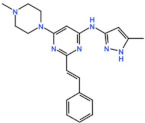	8 Clinical trials	Phase I/II Completed	Soft Tissue Sarcoma, Ovarian Cancer, Triple-Negative Breast Cancer, Relapsed or Refractory Hematological Malignancies, Advanced Fibrolamellar Carcinoma, Multiple Myeloma, Advanced Malignancies	Monotherapy	Best response was PR for ovarian cancer, triple-negative breast cancer, advanced fibrolamellar carcinoma, undifferentiated pleomorphic sarcoma, and angiosarcoma; and CRi for relapsed or refractory hematological malignancies	[150,151,152,153,154,155,156,167]
LY3295668 (Erbumine) 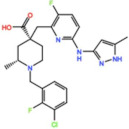	4 Clinical trials	Phase I/II 2 Completed 1 Recruiting 1 Planned	Small-Cell Lung Cancer, Metastatic Breast Cancer, Neuroblastoma, Solid Tumors	Monotherapy Combination with Topotecan and Cyclophosphamide	Best response was SD	[157]
MLN8054 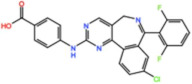	NCT00249301	Phase I Terminated	Solid Tumors	Monotherapy	Best response was SD	[159]
	NCT00652158	Phase I Terminated	Advanced Malignancies	Monotherapy		[160]
MK-5108/VX-689 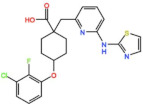	NCT00543387	Phase I Completed	Solid Tumors	Monotherapy	Best response was SD	[162]
				Combination with Docetaxel	Best response was PR	
TAS-119 (Structure Undisclosed)	NCT02448589	Phase I Terminated	Advanced Solid Tumors	Monotherapy	Best response was SD	[164]
	NCT02134067	Phase I Terminated		Combination with Paclitaxel	Best response was PR for ovarian/fallopian tube cancers	[165]
KW-2449 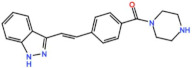	NCT00346632	Phase I Terminated	Acute Leukemias Myelodysplastic Syndromes—Chronic Myelogenous Leukemia	Monotherapy	Terminated due to suboptimal dosing schedule	-
	NCT00779480	Phase I Terminated	Acute Myelogenous Leukemia	Monotherapy	Failure to demonstrate a tolerable dose that had potential for efficacy	

^1^ Data collected from clinicaltrials.gov. ^2^ CR, complete remission; Cri, complete remission with incomplete blood count remission; PR, partial response; SD, stable disease.

In summary, considering all Aurora inhibitors, either those with broad-spectrum or with selective activity, the AEs observed were mainly hematological, primarily neutropenia, which was manageable through G-CSF support. As to pan-Aurora inhibitors, the majority of small molecules were used as monotherapy and demonstrated a poor or modest efficacy in solid and hematological tumors, except for the clinical trials with Cenisertib and Tozasertib, in which CRs were reported in patients with leukemia. CRs in leukemia patients were also observed with Ilorasertib in combination with Azacitidine. Similar efficacy results were observed with the AurB-selective inhibitors. The best responses were achieved in patients with AML with Barasertib as monotherapy and in combination with Cytarabine, while in patients with solid tumors, all inhibitors showed modest efficacy. AurA inhibitors have demonstrated better responses in patients with solid tumors comparatively to AurB and pan-Aurora inhibitors, especially with Alisertib. Even so, the efficacy outcomes of AurA inhibitors were better in hematological tumors than in solid tumors. There is a substantial debate as to whether it is more efficient to inhibit AurA, AurB, or both simultaneously. A pre-clinical study in pancreatic cancer cells has pointed to AurA as a better target than AurB [168]. However, another pre-clinical study has demonstrated that colon cancer cells were more sensitive to AurB inhibition compared to AurA [78]. In fact, the molecule with better efficacy outcomes in trials was the AurA inhibitor Alisertib, but both strategies have demonstrated antitumor activity, especially against hematological tumors. Perhaps, some tumors may be more sensitive to inhibition of one of the two kinases, but further studies are required to address this question. An important aspect of Aurora kinase inhibition is the existence of biomarkers that permit access to its cellular activity, such as histone H3 phosphorylation or autophosphorylation on T288 of AurA, enabling one to verify whether the inhibitors are efficiently targeting the kinases [169]. In sum, either AurA or AurB inhibition seem to be sustainable approaches for cancer therapy that could be improved in combination with other drugs.

## 5. CENP-E Kinesin

Centromere-associated protein E (CENP-E) is a plus end-directed motor protein that plays a crucial role in cytokinesis, chromosome congression and alignment, and in SAC signaling through modulation of BubR1 function [170,171,172]. Inhibition of CENP-E results in mitotic arrest due to unaligned chromosomes, which activates the SAC and has demonstrated antitumor activity in human cancer models [173,174,175]. Some CENP-E inhibitors have been tested in pre-clinical studies, but only one small molecule, GSK923295, has reached clinical trials (Figure 1 and Table 6). GSK923295 is an allosteric inhibitor of CENP-E with an IC_50_ of 1.6 nM in vitro [176]. In the phase I trial (NCT00504790), the MTD was established as 190 mg/m^2^ [177]. GSK923295 was generally well tolerated; the most common AEs were fatigue, diarrhea, and decreased appetite. Five patients (12.8%) experienced DLTs such as increase in aspartate aminotransferase (AST), fatigue, hypoxia, and hypokalemia [177]. Antitumor activity was modest, and the best response was one PR (3%) in a patient with urothelial carcinoma treated with a dose above the MTD (250 mg/m^2^), and 33% had SD [177]. More studies are needed to overcome the challenges of using the CENP-E inhibition approach, in order to develop novel CENP-E inhibitors and to test combinational treatments with other drugs for possible synergistic effects.

**Table 6:** CENP-E inhibitors in clinical trials ^1^.

Compound	Clinical Trials	Current Status	Conditions	Interventions	Outcomes ^2^	Ref.
GSK923295 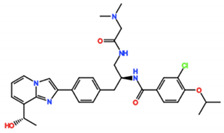	NCT00504790	Phase I Completed	Refractory Cancer	Monotherapy	Best response was PR for urothelial carcinoma	[177]

^1^ Data collected from clinicaltrials.gov. ^2^ PR, partial response.

## 6. Eg-5 Kinesin

Eg-5 kinesin is a plus end-directed motor protein that plays a critical role in bipolar spindle assembly [178,179]. The inhibition of Eg-5 results in monopolar spindles, which leads to SAC activation and mitotic arrest, and has demonstrated antitumor activity in human xenografts models [180,181,182]. Together with its overexpression in several tumors, this makes Eg-5 an attractive target for cancer therapy [183,184,185,186]. Conversely to the kinesin CENP-E, ten Eg-5 inhibitors have reached clinical trials: 4SC-205, ARRY-520 (Filanesib), AZD4877, MK-0731, SB-715992 (Ispinesib), LY2523355 (Litronesib), SB-743921, EMD 534085, ARQ 621, and ALN-VSP02 (Figure 1 and Table 7). Forty-five phase I/II trials against solid and hematological tumors, in monotherapy or in combinational treatments, have been completed or terminated.

### 6.1. SB-715992 (Ispinesib) and SB-743921

Ispinesib, developed by Cytokinetics and GlaxoSmithKline, was the first Eg-5 inhibitor to enter clinical trials. Thirteen studies have been completed or terminated as monotherapy against solid and hematological tumors, and three phase I/II trials in combination with Docetaxel (NCT00169520), Capecitabine (NCT00119171), and Carboplatin (NCT00136578) against solid tumors have also been completed [187,188,189]. In the phase I study in combination with Docetaxel, the MTD was established as 10 mg/m^2^ of Ispinesib and 60 mg/m^2^ of Docetaxel; prolonged neutropenia and febrile neutropenia were the DLTs observed, although the safety profile was considered acceptable and manageable [187]. Similar results were obtained with Carboplatin and Capecitabine, in which the best response was SD, and the DLTs observed were thrombocytopenia and neutropenia, respectively [188,189]. The efficacy outcomes with Ispinesib as monotherapy in patients with liver cancer (NCT00095992), metastatic prostate cancer (NCT00096499), recurrent or metastatic squamous cell carcinoma of the head and neck (NCT00095628), melanoma (NCT00095953), and metastatic kidney cancer (NCT00354250) were also disappointing, with SD being the best response [190,191,192,193,194]. Better results, although modest, were reported from trials against ovarian cancer (NCT00097409) and breast cancer (NCT00607841), in which PRs were observed in 5% and 6.7% of patients, respectively [195,196]. Additionally, a phase I trial on pediatric patients with relapsed or refractory solid tumors has also been initiated with Ispinesib as monotherapy (NCT00363272). Similarly to the previous studies, Ispinesib was well tolerated, but no objective responses were observed, and only three SDs (12.5%) were reported [197].

Meanwhile, another small molecule derived from Ispinesib, SB-743921, was discovered, which exhibited a five-fold increase in potency against Eg-5 compared to Ispinesib, and reached two phase I/II trials, both already completed [198]. In the first-in-human trial (NCT00136513) in patients with solid tumors as monotherapy, the MTD was established as 4 mg/m^2^, and neutropenia was the most common DLT registered. Six patients (15%) showed SD as the best response and a PR was reported in a patient (2.3%) with cholangiocarcinoma [199]. In another phase I/II trial with non-Hodgkin lymphoma and Hodgkin lymphoma patients (NCT00343564), it was reported that DLT and MTD were significantly increased when SB-743921 was co-administered with G-CSF [200]. In this study, four PRs (7.1%) were reported: in three patients with Hodgkin lymphoma, and in one patient with non-Hodgkin lymphoma [200].

### 6.2. ARRY-250 (Filanesib)

Filanesib is a potent Eg-5 inhibitor with an IC_50_ of 6 nM in vitro [201]. Eight phase I/II studies have been completed in patients with solid and hematological tumors. In the phase I trial (NCT00462358) with patients with solid tumors as monotherapy or with G-CSF support (Filgrastim), the MTD was defined as 1.25 mg/m^2^ without prophylactic Filgrastim, and as 1.60 mg/m^2^ in combination with Filgrastim [202]. Filanesib was observed to cause myelosuppression, with the most common treatment-related AEs being febrile neutropenia, neutropenia, leukopenia, and thrombocytopenia [202]. No objective responses were reported and 18% of patients achieved SD as best response [202]. In another monotherapy phase I/II trial in AML patients (NCT00637052), hematological toxicities were the most common AEs. SD was observed in 10% of patients and one PR (3%) was reported [203]. The most promising outcomes were reported in patients with multiple myeloma. The first phase I/II trial with patients with multiple myeloma started in 2009 (NCT00821249) to assess the safety profile and efficacy of Filanesib. Based on the myelosuppression reported in previous studies, G-CSF (Filgrastim) was added to the treatment regimen during phase I and II [204]. In phase II, patients were treated with Filanesib as monotherapy (including G-CSF support), or in combination with low doses of Dexamethasone. As a single agent, 39% of patients had SD and five PRs (16%) were reported, whereas in combination with Dexamethasone, 41% of patients achieved SD and eight PRs (15%) were observed [204]. These results prompted the use of Filanesib in more trials in patients with multiple myeloma in combination with the proteasome inhibitors Bortezomib and Carfilzomib. In a phase I trial (NCT01248923), Filanesib was administrated in combination with Bortezomib and Dexamethasone (including G-CSF support). The safety profile was considered favorable and the hematological toxicities were manageable through G-CSF support [205]. The overall response rate (ORR) was 20%, including one CR (2%) and ten PRs (18%) [205]. In another phase I trial (NCT01372540), Filanesib was administrated in combination with Carfilzomib and Dexamethasone, and G-CSF support was also included. Overall, no CR was reported, although twenty-three PRs (37%) were observed [206]. Additionally, in another phase I/II trial (NCT02384083), Filanesib was combined with Pomalidomide and Dexamethasone, and G-CSF support was also included. Despite the G-CSF support, more than 60% of the patients developed grade 3/4 neutropenia; nevertheless, the efficacy results were promising with an ORR of 51%, including two CRs (4%) and twenty-one PRs (47%) [207]. Considering these good results, Filanesib will likely enter a phase III trial against multiple myeloma.

### 6.3. ALN-VSP02

ALN-VSP02 is unique among the anti-Eg-5 as it is an siRNA lipid nanoparticle formulation targeting the expression of Eg-5 and vascular endothelial growth factor (VEGF) [208]. Two phase I trials were completed with patients with solid tumors as monotherapy (NCT01158079 and NCT00882180). Patients were enrolled sequentially on one of seven dose levels, ranging from 0.1 to 1.5 mg/kg. ALN-VSP02 demonstrated a safety profile at multiple doses, with fatigue, asthenia, nausea, vomiting, and fever being the most common AEs. The best response was a CR (2.7%) observed in a patient with endometrial cancer with multiple hepatic metastases [209].

### 6.4. Litronesib

Litronesib, developed by Kyowa Kirin and Eli Lilly and Company, was demonstrated to inhibit Eg-5 with an IC_50_ of 26 nM in vitro [210]. At least seven phase I/II trials were completed, involving solid tumor patients treated with Litronesib as monotherapy or with G-CSF support (Pegfilgrastim and Filgrastim). Generally, the efficacy results were disappointing. In two phase I trials in advanced tumor patients (NCT01214629 and NCT01214642), Litronesib was administrated as single agent or in combination with Pegfilgrastim. In the first-in-human study with Litronesib (NCT01214629), the MTD was 4 mg/m^2^ for Litronesib without G-CSF support, and 6 mg/m^2^ with Pegfilgrastim. Neutropenia and leukopenia were the most common AEs observed. In total, 26% of patients had SD as the best response, and two PRs (3.7%) were reported in patients with platinum-sensitive ovarian carcinoma and neuroendocrine carcinoma [211], whereas in the other trial (NCT01214642), the safety profile was similar, but no objective responses were observed, with 36.5% of patients achieving SD as the best response [211]. Additionally, another study with Litronesib as monotherapy in patients with solid tumors was initiated in 2011 (NCT01358019). Again, the best response was SD observed in two patients (16.7%) [212]. No further development is planned for this Eg-5 inhibitor, since Kyowa Kirin and Eli Lilly and Company decided to discontinue Litronesib.

### 6.5. EMD 534085

EMD 534085 was developed by Merck-KGaA and was shown to inhibit Eg-5 with an IC_50_ of 8 nM in vitro [213]. EMD 534085 entered a phase I trial in patients with solid tumors or lymphoma as a single agent. The MTD was defined as 108 mg/m^2^ and neutropenia was the most common DLT observed. No objective responses were reported, with 52% of patients achieving SD [214]. No further development is planned with this inhibitor.

### 6.6. SC-205

4SC-205, developed by 4SC, has entered a phase I study against advanced malignancies as monotherapy (NCT01065025). Patients received 4SC-205 once a week or twice weekly. The MTD was defined as 150 mg/m^2^ (once weekly) and 75 mg/m^2^ (twice weekly). Neutropenia was the most common DLT, similarly to the results obtained with the other Eg-5 inhibitors. No CR or PR were reported, and 28% of patients had SD as the best response [215].

### 6.7. AZD4877

AZD4877, from AstraZeneca, inhibits Eg-5 with an IC_50_ of 2 nM in vitro, and has entered six phase I/II trials against solid and hematological tumors [216]. Overall, the results were disappointing. In the phase I trials in solid tumor patients (NCT00613652 and NCT00389389), neutropenia was the most common DLT [217,218]. No objective responses were observed, with 31% and 27% of patients achieving SD, respectively [217,218]. Other trials with AML (NCT00486265) and urothelial cancer (NCT00661609) patients demonstrated similar efficacy. In the phase I trial, all AML patients had treatment failure, whereas in the phase II study with urothelial cancer patients, 18% of patients had SD, and no objective responses were reported [219,220]. No further development is planned for AZ4877.

### 6.8. ARQ 621

ARQ 621, from ArQule, demonstrated a broad spectrum toxicity against a panel of human cancer cell lines [221]. In 2009, ARQ 621 entered a phase I trial with solid tumor patients as monotherapy (NCT00825487). ARQ 621 appeared to be well tolerated at a dose of 280 mg/m^2^, with the most common AEs being fatigue, nausea, and anemia. No objective responses were observed, with 12.5% of patients achieving SD as the best response [222]. No further development is planned for this compound.

### 6.9. MK-0731

MK-0731, developed by Merck Sharp & Dohme, inhibits Eg-5 with an IC_50_ of 2.2 nM in vitro [223]. MK-0731 has entered a phase I trial against advanced solid tumors as monotherapy in 2005 (NCT00104364). The MTD was determined as 17 mg/m^2^, neutropenia being the major DLT observed [224]. No objective responses were reported and only four patients (4.3%) achieved SD [224]. Development of MK-0731 has been halted.

The safety outcomes of the Eg-5 inhibitors were similar to those of Mps1, Plk1, and Aurora kinase inhibitors, with neutropenia as the most common AE. Generally, Eg-5 inhibitors exhibited poor or modest efficacy in patients with solid tumors, either as monotherapy or in combinational treatments, except for a CR observed with ALN-VSP02 as a single agent in a patient with endometrial cancer with multiple hepatic metastases. The most promising efficacy results were achieved with Filanesib in multiple myeloma patients, especially when combined with the proteasome inhibitors Bortezomib and Carfilzomib. It is expected that Filanesib will enter phase III trials. One possible explanation for the promising results of Filanesib is its better pharmacokinetic profile, primarily a higher half-life compared to the other Eg-5 inhibitors. More studies must be performed to assess which drugs demonstrate synergistic effects with Eg-5 inhibitors, as well as to identify novel and more potent inhibitors with a better pharmacokinetic profile, in order to improve the efficacy of the Eg-5 inhibition-based therapeutic approach.

**Table 7:** Eg-5 inhibitors in clinical trials ^1^.

Compound	Clinical Trials	Current Status	Conditions	Interventions	Outcomes ^2^	Refs.
SB-715992 (Ispinesib) 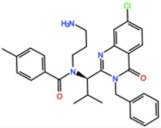	16 Clinical trials	Phase I/II 14 Completed 2 Terminated	Metastatic Prostate, Kidney and Colorectal Cancer, Recurrent or Metastatic Squamous Cell Carcinoma of the Head and Neck, Breast, Ovarian and Liver Cancer, Melanoma, Hodgkin’s or Non-Hodgkin’s Lymphoma, Acute Leukemia Advanced Myelodysplastic Syndromes	Monotherapy	Best response was a PR for ovarian and breast cancer	[190,191,192,193,194,195,196,197]
				Combination with Docetaxel, Capecitabine and Carboplatin	Best response was SD	
SB-743921 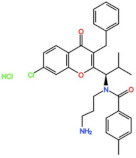	NCT00136513	Phase I Completed	Solid Tumors	Monotherapy	Best response was PR for cholangiocarcinoma	[199]
	NCT00343564	Phase I Phase II Completed	Non-Hodgkin Lymphoma and Hodgkin Lymphoma	Monotherapy	Best response was PR for non-Hodgkin lymphoma	[200]
ARRY-520 (Filanesib) 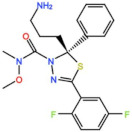	8 Clinical trials	Phase I/II Completed	Multiple Myeloma, Advanced/Refractory Myeloid Leukemia, Advanced Solid Tumors	Monotherapy	Best response was a PR for multiple myeloma	[202,203,204,205,206,207]
				Combination with Pomalidomide, Dexamethasone, Carfilzomib, Filgrastim, Bortezomib, Carfilzomib	Best responses were a CR for multiple myeloma in combination with Pomalidomide, Bortezomib, Dexamethasone, and Filgrastim; PR for multiple myeloma in combination with Bortezomib, Pomalidomide, Dexamethasone, Filgrastim, and Carfilzomib	
ALN-VSP02 (siRNA)	NCT01158079	Phase I Completed	Solid Tumors	Monotherapy	Best response was CR for endometrial cancer with multiple hepatic metastases	[209]
	NCT00882180	Phase I Completed				
LY2523355 (Litronesib) 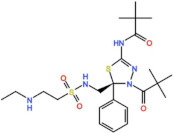	7 Clinical trials	Phase I/II 6 Completed 1 Terminated	Metastatic Breast Cancer, Metastatic and/or Advanced Cancer, Acute Leukemia, Small Cell Lung Cancer	Monotherapy, Combination with Pegfilgrastim, and Filgrastim	Best response was PR for non-small-cell lung cancer, ovarian and neuroendocrine carcinomas, and breast cancer in combination with Pegfilgrastim	[211,212]
EMD 534085 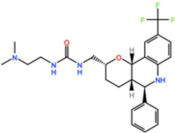	Unknown	Phase I	Advanced Solid Tumors, Lymphoma	Monotherapy	Best response was SD	[214]
4SC-205 (Structure Undisclosed)	NCT01065025	Phase I Completed	Advanced Malignancies	Monotherapy	Best response was SD	[215]
AZD4877 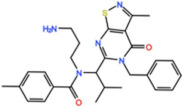	6 Clinical trials	Phase I/II 3 Completed 3 Terminated	Bladder Cancer, Transitional Cell Bladder Cancer, Urethra Cancer, Ureter Cancer, Renal Pelvis Cancer, Acute Myelogenous Leukemia, Non-Hodgkin Lymphoma	Monotherapy	Best response was SD for non-Hodgkin lymphoma, acute myeloid leukemia, and urothelial cancer	[217,218,219,220,225]
ARQ 621 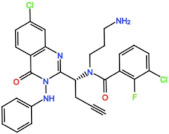	NCT00825487	Phase I Completed	Metastatic Solid Tumors, Refractory/Relapsed Hematologic Malignancies	Monotherapy	Best response was SD	[222]
MK-0731 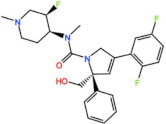	NCT00104364	Phase I Completed	Advanced Solid Malignancies	Monotherapy	Best response was SD for non-small-cell lung cancer, cervical and ovarian cancer	[224]

^1^ Data collected from clinicaltrials.gov. ^2^ CR, complete remission; PR, partial response; SD, stable disease.

## 7. Conclusions and Perspectives

MTAs were the first class of antimitotics that demonstrated clinical benefits in cancer patients. However, several tumors have developed resistance, which, together with the neurological and myeloid toxicity provoked by these agents, has led to the search for and development of alternative approaches to block mitosis. Our increased knowledge on mitotic events has provided an opportunity to identify key mitotic proteins that could be targeted for cancer therapy. As described in this review, several inhibitors, known as second-generation antimitotics (SGAs), were identified for Mps1, Aurora kinases, Plk1, Eg-5, and, to a lesser extent, CENP-E, and these have reached clinical trials.

Among all SGAs tested, the most promising molecules are (i) the Plk1 inhibitors Volasertib and Rigosertib, which demonstrated good efficacy results in AML and MDS patients, respectively; (ii) the AurB inhibitor Barasertib for patients with AML; (iii) the AurA inhibitor Alisertib, which has demonstrated antitumor activity against several solid and hematological tumors; (iv) the pan-Aurora inhibitors Ilorasertib and Cenisertib for patients with AML; and (v) the Eg-5 inhibitor Filanesib for patients with multiple myeloma. However, many AEs were reported. The most common AEs associated with these SGAs were hematological, primarily neutropenia. These results were somehow expected given the high proliferation rate of the bone marrow cells [226]. In the same line of thought, better responses were achieved in patients with hematological tumors than in those with solid cancers, in concordance with the lower proliferation rate observed in solid tumors, compared to hematological cancers [226].

Until now, no small molecule has been approved for clinical use, and MTAs still remain the antimitotic agents with the best therapeutic benefit. While SGAs target individual proteins, MTAs destabilize the huge microtubule/cytoskeleton network involved in the dynamics of several proteins and organelles, thereby indirectly affecting their function. This could be a plausible explanation for the MTAs’ therapeutic success, compared to SGAs. Interestingly, better responses were achieved in combinational treatments than with SGAs as monotherapy. Several studies reported the synergistic effects of different SGAs with many drugs such as MTAs or platinum-based agents. Therefore, combinational treatment represents a solid strategy for achieving better therapeutic effectiveness, while decreasing drug dosage and minimizing AEs and resistance.

From an SAC response point of view, the SGAs are divided into two groups [11,227]. The mitotic blockers, which include the inhibitors of CENP-E, Eg-5, and Plk1, activate the SAC, thereby inducing a prolonged mitotic delay that is expected to culminate in cell death. The mitotic drivers, which include the inhibitors of Mps1 and Aurora B kinase, override the SAC and induce premature mitotic exit with extensive chromosome missegregation, resulting in chromosome aberrations that are incompatible with the cell viability of daughter cells. Thus, it seems reasonable to use both approaches for cancer therapy. Yet, some challenges need to be overcome. There is profound inter- and an intra-variability in terms of cell fates following the prolonged mitotic arrest of cancer cells treated with mitotic blockers [228]. Prolonged mitotic arrest can result in cell death in mitosis, or mitotic slippage (also known as checkpoint adaptation), in which cells exit mitosis without cell division and return to the interphase as tetraploid cells. These cells can undergo cell cycle arrest, die, or re-replicate their genomes and endocycle [228]. Slippage is one of the resistance mechanisms against antimitotic drugs.

On the other hand, mitotic drivers can fuel genomic instability. In case of the incomplete inhibition of mitotic driver targets, which is more likely to occur in vivo than in vitro, chromosome segregation errors may be generated below the threshold required to kill cancer cells, which, theoretically, increases genomic instability, thereby fueling malignancy. This may explain the general failure of both mitotic blockers and mitotic drivers in clinical trials when used as monotherapy. Nonetheless, despite their poor clinical activity as single agents, SGAs may be valuable for synthetic lethal combinations intended to selectively target cancer cells, thus decreasing the risk of mitotic slippage, while enhancing the therapeutic window.

SGAs exhibited promising antitumor activity in pre-clinical studies, but failed in clinical trials. A possible reason for this differential antitumor activity is that two-dimensional (2D) cultures lack cell–cell contacts and a natural tumor microenvironment, which are important in tumor signaling and drug response [229]. Using preclinical models that better mimic the tumor microenvironment, such as patient-derived 3D tumors, should increase the predictive value of pre-clinical antimitotic research, helping in anticipating clinical outcomes [230].

The most common AEs observed in patients treated with SGA were related to hematologic dysfunction. The development of drug-delivery systems could be a valuable approach to overcome this issue, facilitating better targeting towards cancer cells and in-tumor drug retention, while increasing the therapeutic window [231].

Patients with the same tumor type respond differently to the same agents, probably due to different genetic and/or epigenetic modifications that alter the sensitivity to a specific drug [232,233]. Thus, patient stratification using predictive biomarkers, together with an in-depth understanding of the mechanisms by which SGAs kill cancer cells, should pave the way to their effective clinical use.

## Data Availability

Publicly available datasets were analyzed in this study. The data can be found here: [https://clinicaltrials.gov/].
